# Successful Treatment of Severe Gamma-Hydroxybutyric Acid Withdrawal Syndrome With Dantrolene

**DOI:** 10.7759/cureus.16398

**Published:** 2021-07-14

**Authors:** Anna Eveline Röell, Dharmanand Ramnarain, Rama Kamal

**Affiliations:** 1 Intensive Care Medicine, Radboud University Medical Center, Nijmegen, NLD; 2 Intensive Care Medicine, Saxenburgh Medisch Centrum, Hardenberg, NLD; 3 Intensive Care Medicine, Elisabeth-TweeSteden Ziekenhuis, Tilburg, NLD; 4 Psychiatry, GGzE, Eindhoven, NLD

**Keywords:** ghb, detoxification, pharmaceutical ghb, dantrolene, neuroleptic malignant syndrome

## Abstract

The prevalence of gamma-butyrolactone/gamma-hydroxybutyric acid (GBL/GHB) use is increasing. The gravity and number of incidents with this drug are relatively high. A feared complication is addiction and its withdrawal syndrome, which can be life-threatening and is difficult to treat. We present the case of a 31-year-old man, admitted to the ICU because of accidental GBL withdrawal. The patient was tachycardic, sweaty, extremely agitated, and showed signs of psychosis. High doses of benzodiazepines, propofol, sufentanil, and quetiapine could not sedate the patient sufficiently. Dosing with pharmaceutical GHB was challenging due to severe gastric retention. As the patient developed hyperthermia and rhabdomyolysis, signs of a neuroleptic malignant syndrome (NMS), he was treated with dantrolene. After 14 days, the patient was discharged to a psychiatric clinic for further treatment. GHB affects multiple neurotransmitters and chronic use causes the up- or down-regulation of several receptors. During GHB withdrawal, the patient developed a hyperexcitable state, in which there was insufficient gamma-aminobutyric acid (GABA) (the most important inhibiting neurotransmitter) and an abundance of glutamate (the most important excitatory neurotransmitter). High-dose benzodiazepines are often advocated as the first-line treatment, but benzodiazepine resistance has frequently been reported. Therefore, treatment with pharmaceutical GHB is advised. Patients with GHB-withdrawal who have clinical signs of NMS can be treated with dantrolene because it regulates the distorted calcium secretion and affects the serotonin and cholinergic system.

## Introduction

Gamma-butyrolactone (GBL) is a drug that is growing in popularity among young adults and adolescents [1]. GBL is an industrial solvent that is rapidly absorbed and metabolized into its active metabolite gamma-hydroxybutyric (GHB) acid. GHB has an anxiolytic, hypnotic, and euphoric effect [1]. The effect of GBL is 2.5 times stronger than that of GHB because of its biological availability, but it is always metabolized into GHB to have a clinical effect.

The most recent epidemiological data of GHB consumption originates from the Netherlands, where 0.4% of adults reported using GHB in the past year [2,3]. However, the prevalence of GHB use in partying adolescents is 14% and the number of GHB users that are undergoing detox treatment is rising [2-4]. The gravity and number of GHB-related incidents are relatively high. In 2017, 4.7% of all ICU admissions were due to drug intoxication; in 22% of all drug incidents (not only ICU), GHB use was reported [3,5]. Complications of GHB use include addiction, withdrawal, acute toxicity, and a serious risk of fatal overdose [6,7]. GHB withdrawal syndrome is difficult to treat and a high percentage of patients relapse over time [7]. Due to these complications and the presence of pre-existent psychiatric and other diseases, the costs associated with the admission of GHB-intoxicated patients to the ICU are higher than any other patients [3,8].

Symptoms of GHB withdrawal are agitation, tremors, tachycardia, insomnia, delirium, autonomic dysregulation, myoclonus, seizures, rhabdomyolysis, liver and kidney failure, and hallucinations, which can develop into an excited delirium [9]. In particular, the latter can be life-threatening. Symptoms of detoxification can arise within 6 to 76 hours after the cessation of chronic GHB use and can persist for 48 hours to 15 days. Months thereafter, symptoms of delirium, psychosis, and/or depression can persist [10]. With GBL use, withdrawal symptoms can arise earlier and be more severe. It is important to start the treatment of GHB withdrawal immediately: the risk of complications is higher when you wait longer [11].

Treatment of GHB withdrawal has not been properly researched [7,12]. High doses of benzodiazepines are usually advocated, but benzodiazepine resistance in GHB withdrawal is relatively common and pharmaceutical GHB is considered the first-line treatment of GHB detoxification [6,7,13]. Medications that can have an additional effect are baclofen, barbiturates, gabapentin, and propofol. Low-dose antipsychotics can be used with caution because they can cause neuroleptic malignant syndrome (NMS) in patients with GHB withdrawal syndrome [14].

We describe the case of a patient admitted to the ICU because of accidental GBL detoxification. Dantrolene was used to treat hyperthermia and rhabdomyolysis.

## Case presentation

A 31-year-old man was admitted to the orthopedic department because of an ankle fracture that needed surgical treatment (osteosynthesis). Two days after surgery, he was admitted to the ICU because of severe agitation and psychotic symptoms (i.e., agitation, suspiciousness, delusions, and hallucinations). He had a history of GBL and cocaine abuse. His medical history was unremarkable with no use of medication. Physical examination revealed an extremely agitated patient with sinus tachycardia of 130 beats per minute and a temperature of 39.9°C. Initial laboratory tests showed leucocytes 3.7 E9/L, creatinine kinase (CK) 939 IU/L, urea 10.3 mmol/L, and creatinine 106 µmol/L. In the absence of sepsis, hypothyroidism, statin use, or other toxic medication, we diagnosed a GHB withdrawal syndrome based on his history. He had previously kept his GBL use secret and had not used GBL for over 24 hours.

He was treated with high doses of benzodiazepines and antipsychotics. Because of the resulting respiratory depression, he was intubated and mechanically ventilated. Despite high levels of lorazepam 2 mg Q4H, continuous infusion of midazolam 5 mg/hour, propofol 2.9 mg/kg/hour, sufentanil 10 µg/hour, quetiapine 100 mg Q4H, and GHB 2250 mg Q2H, it was difficult to achieve adequate sedation. The concentration of GBL that the patient used at home was calculated using liquid chromatography with tandem mass spectrometry (LC-MS/MS) and was almost 100% pure. We estimated this to be seven times stronger than our pharmaceutical GHB-dosage (150 mg/mL) with an additional 2.5 times higher biological availability of GBL in comparison to GHB. Despite this knowledge, titrating pharmaceutical GHB was challenging due to severe gastric retention in the first five ICU days despite the prescription of prokinetic medication.

Hyperthermia persisted without any evidence of bacterial infection. His temperature rose to 41.5°C and his CK values increased to 33.360 IU/L. Malignant hyperthermia was ruled out in the absence of generalized rigidity. To prevent further deterioration, the patient was cooled therapeutically to temperatures under 39.0°C. Despite cooling and broad-spectrum antibiotics, high levels of CK persisted. Because of suspected quetiapine-induced pyrexia, in combination with possible quetiapine accumulation due to erythromycin-induced CYP3A4 inhibition, the administration of quetiapine and erythromycin was ceased. However, in the following days, hyperthermia and rhabdomyolysis persisted. To treat the persisting hyperthermia and rhabdomyolysis, off-label treatment with dantrolene 4 mg/kg/day was started on day 7. During treatment, the patient’s temperature normalized and CK levels declined dramatically within two days. After four days, dantrolene was stopped and there was no indication for treatment with benzodiazepines, antipsychotics, propofol, and/or sufentanil. The patient woke with no agitation and could be weaned from the ventilator. After 14 days, he was transferred to a psychiatric clinic.

## Discussion

GBL is a GHB precursor. GHB is an endogenous neurotransmitter and neuromodulator that influences neuroprotective mechanisms and deep sleep [15]. Endogenous GHB is made from gamma-aminobutyric acid (GABA), the most important inhibiting neurotransmitter. Exogenous GHB overloads the endogenous GHB system, causing an increase in GABA, reinforcing its inhibiting effect [16].

The use of GHB causes a dose-dependent biphasic effect (Table 1). Initially, there is a stimulant-like effect, followed by a mixture of sedation and a stimulant-like effect. In a low dose, GHB has an additional effect on endogenous GHB. This leads to negative feedback on GABA, thereby causing the drug user to feel euphoric. In higher doses, GHB also stimulates the GHB- and the GABA-A receptor, causing an increase in GABA and therefore a sedating effect [6,7]. It also directly affects the GABA-B receptor, causing an increase in dopamine, thus leading to a stimulating or inhibiting effect depending on the location of the receptor. Chronic use causes GHB tolerance due to lower endogenous GABA and dopamine production and upregulation of dopamine (D1 and D2) receptors. When chronic GHB use is ceased, there is too little GABA to stimulate the desensitized receptors, which causes insomnia and anxiety. A few hours after cessation, the inhibition of dopamine is reversed, causing an acute rise in dopamine concentration. This could lead to psychosis [6,7].

**Table 1:** The effect of exogenous GHB on neurotransmitters and behavior. * R: Receptor ** Neurosteroids are endo- or exogenous steroids that affect the threshold potential of neurons. D1R: Dopamine D1; GABA: Gamma-aminobutyric acid; GBL: gamma-butyrolactone; GHB: gamma-hydroxybutyric acid; GHBR: GHB receptor; NMDAR: N-methyl-D-aspartate receptor.

GHB/GBL use			GHB/GBL detoxification		
Receptor	Neurotransmitter effect	Clinical effect	Chronic use	Neurotransmitter effect	Clinical effect	Treatment
Direct effect						
GHBR	Increased GABA levels	Deep sleep, rewards memory	Tolerance, less endogenous GABA	Shortness of GABA	Insomnia	Barbiturates
GABA-A R*	Increased GABA levels	Sleep, decreased anxiety	Tolerance, less endogenous GABA	Shortness of GABA	Insomnia, anxiety	Benzodiazepines, propofol
GABA-B R	Increased dopamine levels	Addiction, stimulating and inhibiting effects	Tolerance, less endogenous dopamine	Acute rise of dopamine levels	Psychosis, delirium, hypertension, sleeplessness, agitation, paranoia, disorientation, confusion, aggression, hallucinations	Baclofen
Indirect effect						
GHBR	Higher serotonin turnover	Euphoria	Dopamine tolerance	Acute rise of dopamine levels	Psychosis, delirium, hypertension, sleeplessness, agitation, paranoia, disorientation, confusion, aggression, hallucinations	
			Less noradrenaline and acetylcholine	More noradrenaline and acetylcholine	Tremors, miosis, sweating, tachycardia, palpitations, dyspnea, nausea, vomiting, diarrhea, anxiety, restlessness	
	Stimulation of growth hormone	Deep sleep	Unknown	Less growth hormone	Insomnia	
GABA-A R	Less noradrenaline and acetylcholine	Less anxiety	Less noradrenaline and acetylcholine	More noradrenaline and acetylcholine	Tremors, miosis, sweating, tachycardia, palpitations, dyspnea, nausea, vomiting, diarrhea, anxiety, restlessness	
	More neurosteroids**	Less anxiety	Upregulation neurosteroid receptor	No effect of neurosteroids	Anxiety, hyperalgesia	
GABA-B R	Decreased glutamate response of nucleus accumbens	Reward and stimulating effect	Tolerance of GABA-B receptor	Increase glutamate, downregulation of NMDAR	Agitation, restlessness	
	Higher serotonin turnover	Euphoria	Dopamine tolerance	Acute rise of dopamine levels	Psychosis, delirium, hypertension, sleeplessness, agitation, paranoia, disorientation, confusion, aggression, hallucinations	Baclofen
			Less noradrenaline and acetylcholine	More noradrenaline and acetylcholine	Muscle contractions, seizures	B-blockers, dantrolene
	Less noradrenaline and acetylcholine	Less anxiety	Less noradrenaline and acetylcholine	More noradrenaline and acetylcholine	Tremors, miosis, sweating, tachycardia, palpitations, dyspnea, nausea, vomiting, diarrhea, anxiety, restlessness	B-blockers
	Stimulation of growth hormone	Deep sleep	Unknown	Less growth hormone	Insomnia	
D1 R (via NMDAR)	More glutamate in the ventral tegmentum	Addiction, reward memory, stimulating effect	Upregulation of D1 and D2 receptors	Acute rise in dopamine levels	Psychosis, delirium, hypertension, sleeplessness, agitation, paranoia, disorientation, confusion, aggression, hallucinations	Baclofen
			Downregulation of NMDAR	Increase in glutamate levels	Craving	-
	More neurosteroids*	Less anxiety	Upregulation of neurosteroid receptor	No effect of neurosteroids	Anxiety, hyperalgesia	-
Unknown receptor	Decreased activity of the hypothalamic-pituitary-adrenal (HPA) axis, fewer stress hormones, and more oxytocin	Less anxiety + effect on dopamine system	Chronic activation of the HPA axis	More stress hormones and less oxytocin	Anxiety	-
	More endogenous opioid production	Sedation, analgesia	More endogenous opioid release	Less endogenous opioids	Hyperalgesia	Opiates

Indirectly, GHB influences serotonin, growth hormone, glutamate, the cholinergic system, neurosteroids, and endogenous opioids, causing a euphoric, anxiolytic, analgesic, and sedative effect. Chronic use causes the chronic activation of these neurotransmitters, leading to dopamine tolerance, less cholinergic activity, more endogenous opioid release, the upregulation of neurosteroid and dopamine (D1 and D2) receptors, and downregulation of the N-methyl-D-aspartate (NMDA) receptor. The cessation of chronic GHB use, therefore, causes an imbalance of the dopamine-, cholinergic- and endogenous opioid system. Then withdrawal symptoms such as tremors, tachycardia, hypertension, hyperalgesia, hallucinations, and psychosis can arise [6,7].

Current treatment strategies mostly focus on the GABA-A receptor (benzodiazepines). However, recent studies show that the effect of GHB is mostly mediated by the GABA-B receptor and specific GABA-A receptors that are unaffected by benzodiazepines, making patients resistant to this treatment [17]. By targeting other receptors, withdrawal symptoms can be treated. Antipsychotics block dopamine receptors, but these can work counterproductively in the first phase of withdrawal as mentioned earlier. They can cause NMS syndrome by acutely depleting the brain of dopamine [18]. This could also have been the reason for our patient’s hyperthermia and rhabdomyolysis. Patients can also be symptomatically treated with beta-blockers, which antagonize the increased cholinergic activity. The effect of GABA can be lengthened by administering barbiturates and baclofen is a GABA-B receptor agonist, causing dopamine upregulation and glutamate stabilization and thus less anxiety and agitation.

During withdrawal, patients can experience motoric restlessness or seizures due to dysregulation of the serotonin and cholinergic system and calcium streams [6,7,19]. It can even lead to hyperthermia and rhabdomyolysis, clinically resembling NMS or malignant hyperthermia [6,7]. Then, muscle relaxation with dantrolene is one of the appointed treatments [18]. Dantrolene works by inhibiting calcium secretion of the sarcoplasmic reticulum of the musculoskeletal cell, preventing muscle contraction and heat production [20]. Therefore, dantrolene can be used in patients with GHB withdrawal that have clinical signs of NMS.

## Conclusions

The effects of GHB and GHB withdrawal on the brain are very complex, as it influences multiple neurotransmitters. Treatment is complicated and - unlike in our case - should be started immediately. The existing literature promotes high-dose benzodiazepines as a first-line treatment for GHB detoxification. However, because of the high risk of resistance to benzodiazepines, treatment should be focused on the GABA-B receptor with baclofen or pharmaceutical GHB. Antipsychotics could negatively influence the neurological condition of patients and cause NMS. Some GHB withdrawal symptoms resemble that of NMS which can be treated with dantrolene.

## References

[1] Carter LP, Pardi D, Gorsline J, Griffiths RR (2009). Illicit gamma-hydroxybutyrate (GHB) and pharmaceutical sodium oxybate (Xyrem®): differences in characteristics and misuse. Drug Alcohol Depend.

[2] CAM: Risicoschatting Gamma-Hydroxyboterzuur 2011 (2011). Bilthoven.

[3] (2020). Trimbos Instituut. Cijfers drugs: gebruik en trends. https://www.trimbos.nl/kennis/cijfers/cijfers-drugs.

[4] Van Laar M, Van Ooyen-Houben M, Cruts A, Meijer R, Croes E (2016). Nationale Drug Monitor: NDM Annual Report.

[5] NICE Dataregister. https://stichting-nice.nl.

[6] Kamal RM, van Noorden MS, Franzek E, Dijkstra BA, Loonen AJ, De Jong CA (2016). The neurobiological mechanisms of gamma-hydroxybutyrate dependence and withdrawal and their clinical relevance: a review. Neuropsychobiology.

[7] Kamal RM, van Noorden MS, Wannet W, Beurmanjer H, Dijkstra BA, Schellekens A (2017). Pharmacological treatment in γ-hydroxybutyrate (GHB) and γ-butyrolactone (GBL) dependence: detoxification and relapse prevention. CNS Drugs.

[8] van Beusekom I, Bakhshi-Raiez F, de Keizer NF, de Lange DW (2019). The healthcare costs of intoxicated patients who survive ICU admission are higher than non-intoxicated ICU patients: a retrospective study combining healthcare insurance data and data from a Dutch national quality registry. BMC Emerg Med.

[9] Snead OC 3rd, Gibson KM (2005). γ-hydroxybutyric acid. N Engl J Med.

[10] Wojtowicz JM, Yarema MC, Wax PM (2008). Withdrawal from gamma-hydroxybutyrate, 1,4-butanediol and gamma-butyrolactone: a case report and systematic review. CJEM.

[11] Kamal RM, Dijkstra BA, Loonen AJ, De Jong CA (2016). The effect of co-occurring substance use on gamma-hydroxybutyric acid withdrawal syndrome. J Addict Med.

[12] von Theobald L, Rousselet M, Cholet J, Debar H, Boels D, Victorri-Vigneau C, Grall-Bronnec M (2017). Inpatient gamma-hydroxybutyrate detoxification: a case report describing day-to-day therapeutic management. J Addict Med.

[13] van Noorden MS, Kamal RM, Dijkstra BA, Mauritz R, de Jong CA (2015). A case series of pharmaceutical gamma-hydroxybutyrate in 3 patients with severe benzodiazepine-resistant gamma-hydroxybutyrate withdrawal in the hospital. Psychosomatics.

[14] Eiden C, Capdevielle D, Deddouche C, Boulenger JP, Blayac JP, Peyrière H (2011). Neuroleptic malignant syndrome-like reaction precipitated by antipsychotics in a patient with gamma-butyrolactone withdrawal. J Addict Med.

[15] Mamelak M, Black J, Montplaisir J, Ristanovic R (2004). A pilot study on the effects of sodium oxybate on sleep architecture and daytime alertness in narcolepsy. Sleep.

[16] Muller C, Viry S, Miehe M, Andriamampandry C, Aunis D, Maitre M (2002). Evidence for a γ-hydroxybutyrate (GHB) uptake by rat brain synaptic vesicles. J Neurochem.

[17] van Nieuwenhuijzen PS, McGregor IS, Chebib M, Hunt GE (2014). Regional Fos-expression induced by γ-hydroxybutyrate (GHB): comparison with γ-butyrolactone (GBL) and effects of co-administration of the GABAB antagonist SCH 50911 and putative GHB antagonist NCS-382. Neuroscience.

[18] van Waarde JA, Muller ME, Verweg B (2006). [Neuroleptic malignant syndrome: a life-threatening complication that can be treated effectively]. Ned Tijdschr Geneeskd.

[19] Coune P, Taleb O, Mensah-Nyagan AG, Maitre M, Kemmel V (2010). Calcium and cAMP signaling induced by gamma-hydroxybutyrate receptor(s) stimulation in NCB-20 neurons. Neuroscience.

[20] Muehlschlegel S, Sims JR (2009). Dantrolene: mechanisms of neuroprotection and possible clinical applications in the neurointensive care unit. Neurocrit Care.

